# Supplementary material for: Feasibility and Acceptability of Personalized Breast Cancer Screening (DECIDO Study): A Single-Arm Proof-of-Concept Trial
Source: Int J Environ Res Public Health. 2022 Aug 21;19(16):10426. doi: 10.3390/ijerph191610426 (PMC9407798; doi:10.3390/ijerph191610426)
Supplement: Supplementary file 1 [file ijerph-19-10426-s001.zip › ijerph-1749956-supplementary.pdf]

Table S1 Breast cancer screening recommendations according to the absolute risk of breast cancer at 5 years

Age group (years)	Absolute risk of breast cancer at 5 years	Screening recommendations
40-44	< 0.99%	Watch and wait
	0.99%-1.16%	Biennial
	> 1.16%	Annual
45-48	< 0.99%	Watch and wait
	0.99%-1.19%	Biennial
	> 1.19%	Annual
49-50	< 0.8%	Triennial
	0.8%-1.19%	Biennial
	> 1.19%	Annual
40-50	> 6%	Referral to the hospital breast unit and/or genetic counseling

Absolute risk of breast cancer at 5 years for average women in Catalonia (Spain) aged 45 years: 0.8%; 50 years: 0.99%; 60 years: 1.16%; 65 years: 1.19%.

Source: Pons-Rodriguez A, Forné Izquierdo C, Vilaplana-Mayoral J, Cruz-Esteve I, Sánchez-López I, Reñé-Reñé M, et al. Feasibility and acceptability of personalised breast cancer screening (DECIDO study): Protocol of a single-arm proof-of-concept trial. *BMJ Open* **2020**;*10*. doi:10.1136/bmjopen-2020-044597
